# Origin‐Specific Adhesive Interactions of Mesenchymal Stem Cells with Platelets Influence Their Behavior After Infusion

**DOI:** 10.1002/stem.2811

**Published:** 2018-03-23

**Authors:** Lozan Sheriff, Asma Alanazi, Lewis S. C. Ward, Carl Ward, Hafsa Munir, Julie Rayes, Mohammed Alassiri, Steve P. Watson, Phil N. Newsome, G. E. Rainger, Neena Kalia, Jon Frampton, Helen M. McGettrick, Gerard B. Nash

**Affiliations:** ^1^ Institute for Cardiovascular Sciences, University of Birmingham, Birmingham United Kingdom; ^2^ Institute of Inflammation and Ageing, University of Birmingham, Birmingham United Kingdom; ^3^ Institute of Cancer and Genomic Sciences, University of Birmingham, Birmingham United Kingdom; ^4^ Centre for Liver Research, Institute of Immunology and Immunotherapy, College of Medical and Dental Sciences, University of Birmingham, Birmingham United Kingdom; ^5^ Medical College, King Saud bin Abdulaziz University for Health Sciences Riyadh KSA; ^6^ National Institute for Health Research, Liver Biomedical Research Unit at University Hospitals Birmingham NHS Foundation Trust and the University of Birmingham Birmingham United Kingdom; ^7^ Liver Unit, University Hospitals Birmingham NHS Foundation Trust Birmingham United Kingdom

**Keywords:** Mesenchymal stem cells, Platelets, Blood, Cell adhesion, Collagen, Umbilical cord, Bone marrow

## Abstract

We investigated the adhesive behavior of mesenchymal stem cells (MSC) in blood, which might influence their fate when infused as therapy. Isolated human bone marrow MSC (BMMSC) or umbilical cord MSC (UCMSC) adhered efficiently from flow to the matrix proteins, collagen, or fibronectin, but did not adhere to endothelial selectins. However, when suspended in blood, BMMSC no longer adhered to collagen, while UCMSC adhered along with many aggregated platelets. Neither MSC adhered to fibronectin from flowing blood, although the fibronectin surface did become coated with a platelet monolayer. UCMSC induced platelet aggregation in platelet rich plasma, and caused a marked drop in platelet count when mixed with whole human or mouse blood in vitro, or when infused into mice. In contrast, BMMSC did not activate platelets or induce changes in platelet count. Interestingly, isolated UCMSC and BMMSC both adhered to predeposited platelets. The differences in behavior in blood were attributable to expression of podoplanin (an activating ligand for the platelet receptor CLEC‐2), which was detected on UCMSC, but not BMMSC. Thus, platelets were activated when bound to UCMSC, but not BMMSC. Platelet aggregation by UCMSC was inhibited by recombinant soluble CLEC‐2, and UCMSC did not cause a reduction in platelet count when mixed with blood from mice deficient in CLEC‐2. We predict that both MSC would carry platelets in the blood, but their interaction with vascular endothelium would depend on podoplanin‐induced activation of the bound platelets. Such interactions with platelets might target MSC to damaged tissue, but could also be thrombotic. Stem Cells *2018;36:1062–1074*

**Significance Statement**

Mesenchymal stem cells (MSC) may be used to for therapy by injection into the blood. However, little is known about the behavior of MSC in flowing blood and subsequent adhesion in blood vessels. This study shows that the adhesive behavior of flowing MSC depends on their origin because this influences how they interact with platelets also found in the blood. MSC from umbilical cords induce platelet activation which could cause clots to form. The platelets also influence how all MSC stick to surfaces, which could affect their deposition in damaged tissues. The results have implications for therapeutic infusion of MSC.

## Introduction

Mesenchymal stem cells (MSC) are multipotent stem‐like cells extracted first from the bone marrow (BM), but recently also obtained from umbilical cords (UCs) and adipose tissue 1. MSC have the ability to repair damaged tissue 2. For instance, administration of bone‐marrow mesenchymal stem cells (BMMSC) improved the outcome in animal models of chronic cardiac ischemia and acute myocardial infarction 3, 4, 5. The mechanisms underlying the therapeutic benefits of MSC are not well understood, but it is believed that MSC promote tissue growth and wound healing, and modulate immune responses 6, 7. Immunosuppressive effects of MSC include induction of apoptosis of activated T cells 8, expansion of immunosuppressive Tregs 9, 10, suppression of monocyte differentiation into dendritic cells 11, and cross‐talk with endothelial cells to downregulate recruitment of leukocytes 8.

Consequently, MSC have been widely used in clinical trials, in which they are commonly administered by intravenous injection 12. The recruitment of circulating MSC into the target tissue remains poorly understood, but may be critical for therapeutic efficacy 13. When delivered systemically, MSC have been found at sites of injury, suggesting that circulating MSC may target damaged tissue 14. However, MSC have a larger diameter than the pulmonary capillaries, which may cause trapping in the lungs and prevent access to the intended organs 15. Nevertheless, it has been suggested that like leukocytes, MSC go through a multistep process to cross the endothelium 16, with initial capture supported by P‐selectin 17. However, we and others have only observed attachment of MSC to endothelial cells from flow at very low shear stress 14, 18. Several studies indicate that platelets could play a role in MSC recruitment. Intravascular infused MSC were observed in contact with neutrophil‐platelet clusters in inflamed ear dermis 19. In a rat model of pulmonary arterial hypertension, MSC deposition in the lung was dependent on platelet activation and mediated by P‐selectin and platelet glycoprotein GPIIb/IIIa (αIIbβ3‐integrin) 20. It appears, therefore, that MSC are not efficient at binding to intact endothelium under circulatory conditions. However, their ability to bind to exposed matrix proteins under flow and their adhesive behavior in flowing whole blood have not been directly analyzed. Moreover, the studies outlined above have all used BMMSC and may not reflect the behavior of MSC from other tissues.

The foregoing suggests that to understand the fate of MSC in the circulation and optimize their therapeutic delivery, we need to define their ability to adhere to different surfaces in the presence of whole blood and the possible effects of tissue origin on these interactions. To answer these questions, BMMSC or umbilical cord mesenchymal stem cells (UCMSC) were suspended in culture medium or in whole blood, perfused through glass capillaries coated with chosen adhesive substrates and their adhesion assessed. We also investigated MSC interactions with platelets in suspension (either purified platelets or whole blood) and as a monolayer deposited on a fibronectin. Finally, we investigated the fate of MSC infused into mice and their effects on platelet numbers in blood. We observed differences in adhesive properties between BMMSC and UCMSC, and especially in their interactions with platelets. This was linked to differential expression of podoplanin, a membrane glycoprotein expressed on some stromal cells as well as lymphatic endothelial cells, and known to activate platelets through its ligand CLEC‐2 21, 22, 23, 24, 25. This study shows that the origin of MSC and differences in their adhesive behavior may have an impact on their therapeutic use.

## Methods

### Ethics and Collection of Tissues

The study was conducted in compliance with the Declaration of Helsinki. All human samples were obtained with written, informed consent and approval from the Human Biomaterial Resource Centre (Birmingham, U.K.), the West Midlands and Black Country Research Ethics Committee, or University of Birmingham Local Ethical Review Committee. All animal experiments were performed under a Home Office Project License in accordance with UK legislation.

Blood was drawn by venepuncture from healthy adult volunteers into citrate phosphate dextrose adenine (CPDA) solution (1:9, blood vol/vol, Sigma‐Aldrich, Poole, UK; https://www.sigmaaldrich.com). Blood was drawn from mice via cardiac puncture and anticoagulated with CPDA. UCs were collected from anonymous donors with the assistance of the Birmingham Women's Health Care NHS Trust and Sandwell and West Birmingham Hospitals NHS Trust.

### Isolation, Culture, and Characterization of MSC

The methods of MSC isolation and culture were as described recently 26, 27. Human BMMSC were purchased from Lonza and cultured in the manufacturer's recommended medium (Mesenchymal Stem Cells Growth Medium BulletKit, Lonza, Ltd., Burton on Trent, UK; https://www.lonza.com/) and used between passages 5 and 10. UCMSC were isolated from UCs as previously described 26 and used between passages 3 and 10. For adhesion assays, MSC were dissociated by trypsin EDTA solution, counted using a Cellometer (Nexcelom Bioscience, Ltd., Lawrence, MA; https://www.nexcelom.com) and suspended at 5 × 10^5^/ml in culture medium.

Cells were identified as MSC based on the criteria of the International Society for Cell Therapies 28. Their ability to differentiate into osteocytes, adipocytes, and chondrocytes, their surface expression of CD44, CD73, CD90, CD105, and CD146, and their lack of expression of CD14, CD20, CD34, and CD45 were verified as recently described 26. To test expression of β1‐, β2‐, and β3‐integrins, MSC were incubated with fluorescein isothiocyanate (FITC)‐conjugated anti‐β_1_‐integrin (Clone B‐D15; Abcam, Cambridge, UK; https://www.abcam.com) or phycoerythrin (PE)‐conjugated anti‐β2‐integrin (Clone 212701; R&D Systems, Abingdon, UK; https://www.rndsystems.com), or with unconjugated anti‐β3‐integrin (SZ21; Beckman Coulter, Inc., High Wycombe, UK; https://www.beckmancoulter.com) followed by incubation with FITC‐conjugated goat anti‐mouse secondary antibody (Dako, Santa Clara; https://www.agilent.com/). Expression of podoplanin (PDPN) was analyzed using PE‐conjugated anti‐podoplanin (clone NZ‐1.3, eBioscience, Waltham, MA; https://www.thermofisher.com). Control samples were incubated with isotype‐matched nonspecific antibodies, either conjugated appropriately or with secondary labeling as above. The level of expression was evaluated using a Cyan ADP flow cytometer. Data were analyzed offline using Summit 4.3 (both Beckman Coulter, Inc.). In some experiments, we fractionated MSC based on the level of expression of podoplanin (i.e., PDPN+ and PDPN− cells) using a MoFlow Astrios cell sorter (Beckman) and returned them to culture. Separated fractions, or fractions within the original population (gated using PDPN expression), were also reanalyzed for expression of MSC markers.

### Adhesion of Flowing MSC to Purified Receptors or Platelets

Adhesion of flowing MSC to chosen substrates was analyzed as recently described 8. Microslides (glass capillaries with rectangular cross‐section of width *W* = 3.0 mm by depth *D* = 0.3 mm) were coated by incubation for 2 hours at 37°C with required proteins in phosphate‐buffered saline (PBS): 1% bovine serum albumin (BSA), 10 μg/ml P‐selectin; 100 μg/ml E‐selectin; 20 μg/ml plasma fibronectin (all from Sigma); 500 µg/ml equine tendon collagen (Horm collagen; Axis‐Shield, Dundee, U.K., https://www.axis-shield.com/). Microslides were flushed with 1% BSA and incubated overnight at 4°C to block nonspecific protein binding sites.

Microslides were attached to a perfusion system and mounted onto the stage of a phase‐contrast and fluorescence video‐microscope, and events recorded as described 8, 27. Cell‐free buffer or MSC were drawn through the microslide at chosen flow rate (*Q*) and hence wall shear rate (*γ*_w_), calculated from the equation *γ*_w_ = 6*Q*/(*W D* ^2^). UCMSC or BMMSC at 5 × 10^5^/ml were flowed over the different surfaces for 4 minutes. The MSC were either suspended in culture medium (as described above) or in whole blood. In the latter case, MSC in culture medium (1 ml at 5 × 10^5^/ml) were centrifuged, the supernatant removed and the cell pellet resuspended in 1 ml blood. When using blood, MSC were first stained with Cell Tracker Green (5 µM; Life Technologies, Waltham MA; https://www.thermofisher.com). Nonadherent cells were washed from the microslide using cell‐free medium and video recordings were made of a series of microscope fields along the centerline of the microslide and analyzed offline. Adherent cells were counted and converted to a percentage of all MSC perfused to obtain a value for efficiency of attachment corrected for flow rate and hence number perfused, as described previously 8.

To assess MSC binding to immobilized platelets, whole blood was perfused through a microslide coated with fibronectin for 4 minutes at a wall shear rate of 35 s^−1^. The microslide was then washed out with PBS without Ca^2+^ and Mg^2+^ so that a platelet monolayer was left on the surface. When desired, to fully activate the deposited platelets, 10 μM thrombin receptor activating peptide (TRAP; Abcam) was then added for 30 minutes.

In some experiments, MSC were pretreated with function‐blocking antibodies against adhesion receptors or antibodies against platelet‐receptors were added to the blood for 10 minutes at room temperature at a concentration of 10 µg/ml before perfusion. Antibodies were against: β1‐integrin (mab13; BD Pharmingen, Wokingham, UK; https://www.bdbiosciences.com/); β3‐integrin (SZ21; Beckman Coulter); αIIbβ3‐integrin (Abciximab; Eli Lilly: Hampshire, U.K., https://www.lilly.co.uk/); GPIb (SZ1; Beckman Coulter).

### Aggregation of Platelets Induced by MSC

Platelet‐rich plasma (PRP) was harvested from blood centrifuged at 300*g* for 5 minutes and platelet‐poor plasma (PPP) after further centrifugation at 1,500*g* for 15 minutes. Seventy microliter of BMMSC or UCMSC at a concentration of 1.5 × 10^6^/ml was added to 280 µl of PRP and mixed for 20 minutes on a roller mixer in a 37°C incubator. The samples were analyzed using a spectrophotometer (Biotec, Bridport, Dorset, U.K., https://biotec.com/) which measured the light absorbance (or turbidity) at 540 nm of PRP (PRPabs) and platelet‐poor plasma (PPPabs), compared to PRP mixed with MSC (MSCabs). In general, the absorbance of PRP decreases when the platelets aggregate, with a minimum being absorbance of PPP. Here, effects of MSC were quantified as absorbance relative to PRP, calculated as: (MSCabs − PPPabs)/(PRPabs − PPPabs) × 100%. Thus, an absorbance of 100% would indicate no effect of the MSC, while reduction would signify increasing aggregation, down to a relative absorbance of 0% (equal to PPP and thus equivalent to removing all platelets). In some experiments, UCMSC were pretreated with antibody against β1‐integrin, or antibody against α_IIb_β_3_‐integrin was added to PRP, or human recombinant CLEC‐2 protein (30 µg/ml; R&D systems, Abingdon, U.K.) was added to MSC and PRP.

### Effect of MSC on Platelet Count in Blood

BMMSC or UCMSC (2.5 × 10^5^) were centrifuged at 400*g* for 5 minutes and the pellet resuspended in 1 ml of human or murine blood and placed on a roller mixer at 37°C for 20 minutes, along with an aliquot of whole blood. Platelet counts were obtained with an ABX Pentra 60 (ABX Diagnostics, Irvine, CA, https://www.horiba.com/). Blood smears were made and stained with hematoxylin and eosin (H&E; Leica, Milton Keynes, UK; https://www.leicabiosystems.com) and examined by light microscope. We compared blood from 8 to 12‐week‐old platelet‐specific CLEC‐2 deficient mice, *Clec1b^fl/fl^*PF4‐Cre, and their matched controls, *Clec1b^fl/fl^* 8.

### MSC Infusion into Mice—Localization and Effects on Platelet Count

Wild‐type male and female C57/BL6 mice at the age 8–12 weeks were obtained from the Charles Rivers Laboratories (Margate, U.K.; https://guide.labanimal.com/supplier/charles-river-uk-ltd). CLEC‐2 deficient mice and matched controls were as described above. BMMSC or UCMSC (2.5 × 10^5^) were suspended in 200 µl of PBS and injected into the tail vein. Control mice received 200 µl of PBS. Mice were bled via the tail into CPDA for platelet counts 24 hours before MSC injection, and 4, 24, 48, and 72 hours postinjection. The final bleed was obtained by cardiac puncture following neck dislocation on isoflurane anesthetized animals. Platelet counts were measured using an ABX Pentra 60.

Alternatively, isolated BMMSC or UCMSC were labeled with 0.3 mg/ml Xenolight DiR (Caliper Life Sciences, Preston Brook, UK, https://www.caliperLS.com) for 30 minutes at 37°C. Cells were washed three times in PBS and resuspended in 200 µl of PBS for tail vein injection. Mice were culled 1‐hour post MSC injection and organs were isolated and imaged using an “IVIS Spectrum In Vivo Imaging System” (Caliper Life Sciences). Images were acquired by Epi‐illumination at 745 nm excitation and 800 nm emission.

### Statistical Analysis

Data are shown as mean ± SEM of (*n*) replicate experiments. Statistical analysis was performed using Minitab 17 software (Minitab, Inc., State College, PA). Effects of multiple conditions were analyzed using analysis of variance and post hoc comparisons between treatments were made using Bonferroni test. Single treatments were compared to control by paired or unpaired *t* test as appropriate.

## Results

### Comparison of Adhesion of UCMSC and BMMSC from Flow

**Figure 1:**
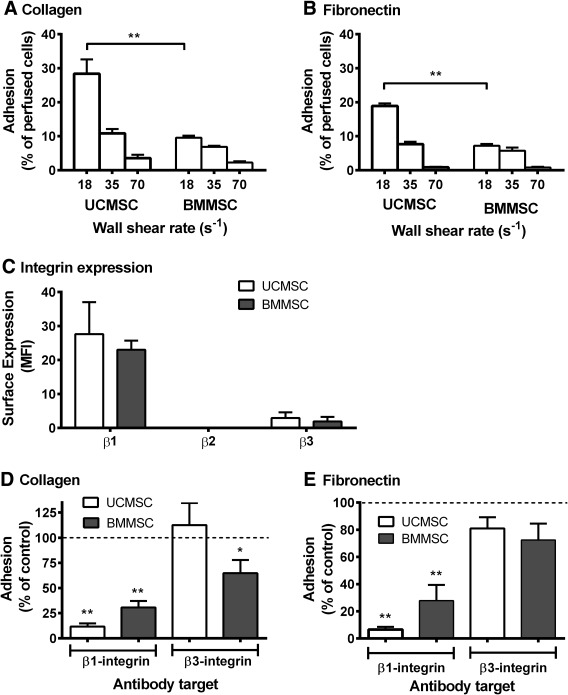
Adhesion of flowing UCMSC and BMMSC to collagen or fibronectin: effect of wall shear rate and roles of integrins. **(A, B):** UCMSC and BMMSC were perfused for 4 minutes over (A) collagen or (B) fibronectin at wall shear rates of 18, 35, or 70 s^−1^. In (A, B), analysis of variance showed significant effects of wall shear rate and of cell type (*p* < .01 in both cases). **, *p* < .01 by Bonferroni test. Data are mean ± SEM from three experiments. **(C):** Expression of β1‐, β2‐, and β3‐integrins was assessed on BMMSC and UCMSC by flow cytometry and expressed as MFI. Data are mean ± SEM from eight experiments (UCMSC) or four experiments (BMMSC), except for β2‐integrins when two isolates of each type were analyzed. (**D, E):** UCMSC and BMMSC were treated with blocking antibodies against β_1_‐ or β_3_‐integrin prior to perfusion over collagen (D) or fibronectin (E) for 4 minutes at a wall shear rate of 35 s^−1^. Data are expressed as adhesion as a percentage of untreated mesenchymal stem cells control. Data are mean ± SEM from three experiments (UCMSC) or six experiments (BMMSC). *, *p* < .05; **, *p* < .01 for comparison to untreated control by paired *t* test. Abbreviations: BMMSC, bone‐marrow mesenchymal stem cells; MFI, median fluorescence intensity; MSC, mesenchymal stem cells; UCMSC, umbilical cord mesenchymal stem cells.

UCMSC and BMMSC were perfused over surfaces coated with receptors that would be presented by activated platelets or inflamed endothelium (P‐selectin and E‐selectin) or by damaged vessel wall (collagen and fibronectin), or with albumin only. Adhesion to P‐selectin, E‐selectin, or albumin was barely detectable; even at a low wall shear rate of 18 s^−1^, <0.1% of perfused cells adhered. When UCMSC were flowed over collagen at a wall shear rate of 18 s^−1^, around 30% adhered (Fig. 1A). Adhesion decreased with increasing shear rate (Fig. 1A). Adhesion of UCMSC to fibronectin showed a similar trend but was, on average, lower than to collagen (Fig. 1B). When BMMSC were perfused over the same surfaces, similar shear‐dependence of adhesion was observed, but overall, adhesion was lower than that seen for UCMSC (Fig. 1). Doubling the highest shear rate to 140 s^−1^ essentially abolished adhesion for either cell type.

### Receptors Underlying MSC Adhesion to Matrix Proteins

We first evaluated levels of expression of β1‐, β2‐, or β3‐integrins. UCMSC and BMMSC showed high levels of expression of β_1_‐integrins, low levels of β_3_‐integrins, and no detectable expression of β_2_‐integrins with no significant differences in expression between the cell types (Fig. 1C).

Next, we tested the effects of pretreating the MSC with function‐blocking antibodies against β_1_‐ or β_3_‐integrins on their adhesion to collagen and fibronectin. UCMSC adhesion to collagen was reduced by ∼90% when β_1_‐integrin was blocked, while blocking β_3_‐integrins had no significant effect (Fig. 1D). Adhesion of BMMSC was decreased by ∼70% when β_1_‐integrin was blocked, while blocking β_3_‐integrins reduced adhesion by ∼35% (Fig. 1D). Overall, β_1_‐integrins supported adhesion of MSC to collagen, with a contribution from β_3_‐integrins for BMMSC. Adhesion of UCMSC to fibronectin was inhibited almost completely by blockade of β_1_‐integrins, but antibody against β_3_‐integrins had no significant effect (Fig. 1E). For BMMSC, blockade of β_1_‐integrins was again much more effective than blockade of β_3_‐integrins (Fig. 1E). Thus, β_1_‐integrins were also the dominant receptors for adhesion to fibronectin.

### Adhesion of MSC from Flowing Whole Blood to Matrix Proteins and to Platelets

**Figure 2:**
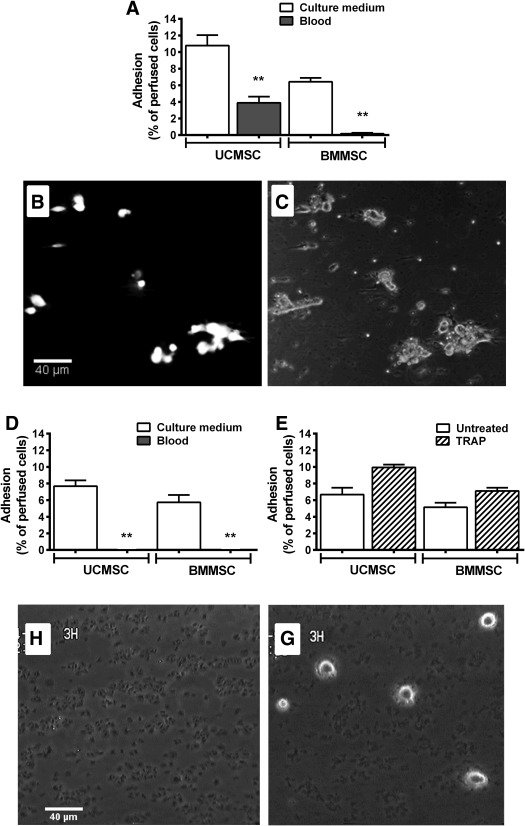
Adhesion of mesenchymal stem cells (MSC) from flowing blood to collagen and fibronectin and to deposited platelets. UCMSC or BMMSC were suspended in blood or in culture medium, and perfused over collagen or fibronectin‐coated surfaces at a wall shear rate 35 s^−1^ for 4 minutes. **(A):** Adhesion to collagen. Analysis of variance (ANOVA) showed a significant effect of cell type and of suspending medium on binding to collagen (both *p* < .01). **(B, C):** Fluorescence and phase contrast micrographs of the same field of view showing clumps of UCMSC labeled with cell tracker green, bound on a collagen surface after perfusion and washout of blood. **(D):** Adhesion to fibronectin. ANOVA showed a significant effect of suspending medium on binding to fibronectin (*p* < .01). **(E):** Adhesion of isolated BMMSC or UCMSC to platelet monolayers which had previously been deposited from blood onto fibronectin, with or without subsequent treatment of the platelets with TRAP. ANOVA showed a significant effect of cell type and of TRAP on adhesion (*p* < .01 in each case). **(F):** Phase contrast micrograph of the platelet monolayer deposited onto fibronectin after perfusion and washout of blood. **(G):** Phase contrast micrograph of UCMSC adhered from flow on a platelet monolayer previously deposited on fibronectin. Data are mean ± SEM from three experiments, except in (A) where adhesion from flowing blood was tested in four experiments. **, *p* < .01 compared to respective MSC tested in culture medium by Bonferroni test. Abbreviations: BMMSC, bone‐marrow mesenchymal stem cells; TRAP, thrombin receptor activating peptide; UCMSC, umbilical cord mesenchymal stem cells.

We added fluorescently labeled MSC to whole blood and perfused the suspension over coated surfaces at a wall shear rate of 35 s^−1^ (the middle value of those previously tested). Interestingly, while many UCMSC in whole blood adhered on collagen (albeit less efficiently than when perfused in culture media), very few BMMSC bound (Fig. 2A). On closer observation, UCMSC adhered in clumps (Fig. 2B). After washing out the blood, platelet “thrombi” were observed on the collagen and around the clumps of UCMSC (Fig. 2C). No MSC adhered to fibronectin when they were perfused in whole blood (Fig. 2D). After washing out the blood, the fibronectin surface was covered by spread platelets in a monolayer without aggregates of “thrombi” (Fig. 2F). It appeared that those platelets inhibited the MSC from binding to the fibronectin itself. However, when isolated MSC were perfused over this platelet‐coated fibronectin, they were able to adhere in large numbers with the percentage of UCMSC adhering being significantly higher than BMMSC (Fig. 2E, 2G). Since these platelets were presumably not fully activated as they did not form aggregates from blood, TRAP was perfused to fully activate them, and then isolated MSC were perfused. TRAP increased the adhesion of both MSC types compared to the untreated platelet monolayers (Fig. 2E).

### Activation and Aggregation of Platelets by MSC in Suspension

The results above showed that MSC were intrinsically able to adhere to matrix proteins and to deposited platelets. However, in blood, this adhesion was effectively “masked,” except when UCMSC adhered to collagen along with activated platelets. We hypothesized that UCMSC adhered to platelets and activated them in blood, while BMMSC could bind but not activate platelets.

**Figure 3:**
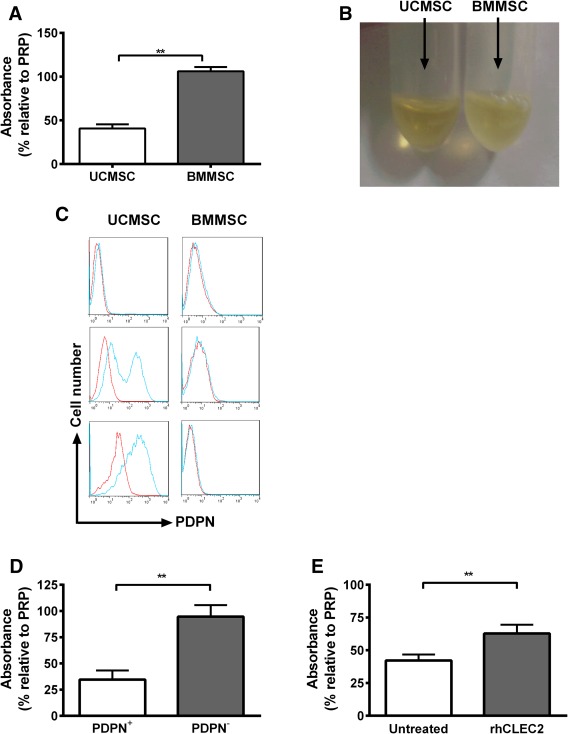
Differential effects of mesenchymal stem cells on platelet aggregation and role of podoplanin. **(A):** UCMSC or BMMSC were mixed with PRP for 20 minutes at 37°C. Platelet aggregation was quantified by a decrease in absorbance compared to PRP. **(B):** Representative images of PRP after mixing with UCMSC or BMMSC, with UCMSC decreasing the turbidity compared to BMMSC. **(C):** UCMSC and BMMSC were stained with an anti‐podoplanin (PDPN, blue) antibody or nonspecific control (red) and analyzed by flow cytometry. Different levels of expression of PDPN were observed on UCMSC, while no expression was detected on BMMSC. **(D):** PDPN positive (PDPN+) or negative (PDPN−) UCMSC were stirred with PRP and platelet aggregation was quantified by a decrease in absorbance compared to PRP alone. **(E):** PRP was either untreated or treated with recombinant human CLEC2 (rhCLEC2) prior to stirring with UCMSC and aggregation was quantified by a decrease in absorbance compared to PRP. Data are mean ± SEM from 6 (A), 4 (B), or 10 (C) experiments. **, *p* < .01 by unpaired *t* test (A, D) or paired *t* test (E). Abbreviations: BMMSC, bone‐marrow mesenchymal stem cells; PRP, platelet‐rich plasma; UCMSC, umbilical cord mesenchymal stem cells.

We thus assessed the ability of MSC to activate and aggregate platelets directly by quantifying the change in absorbance (or turbidity) of PRP after UCMSC or BMMSC were added. In general, the absorbance of PRP decreases when platelets become activated and aggregate, toward a minimum absorbance measured for PPP. We found that absorbance relative to PRP decreased dramatically when UCMSC were mixed with PRP, but not when BMMSC were added (Fig. 3A), indicating that only the UCMSC induced aggregation. The effect is illustrated in Figure 3B, which shows the turbid appearance of PRP mixed with BMMSC, compared to the relative clarity of PRP mixed with UCMSC.

Some stromal cells express podoplanin which can activate platelets through their membrane receptor CLEC‐2 25, 28. On screening a number of isolates of UCMSC and BMMSC, we found that BMMSC did not express podoplanin but UCMSC usually did (see, e.g., Fig. 3C). Among 14 UCMSC isolates, 3 lacked expression and 11 showed positive expression (of which 3 had bimodal distributions). Considering the immunophenotype of UCMSC, all the aforementioned isolates meet the criteria of the International Society for Cell Therapies to be identified as MSC based on expression of MSC markers. Bimodal isolates were subsequently sorted into podoplanin positive and negative fractions and analyzed. Podoplanin status following cell sorting was very stable, even after freezing in liquid nitrogen, thawing and reculture. Moreover, expression of CD44, CD90, and CD73 and differentiation capacity were essentially identical for positive and negative cells when reanalyzed after fractionation (for three different isolates), or after gating on podoplanin for unfractionated bimodal isolates (two isolates). We also found that all isolates could be differentiated regardless of podoplanin expression. While expression of podoplanin might have functional significance (see below), it did not appear to identify different “stemness” among cells.

We thus tested effects of podoplanin expression on platelet aggregation. UCMSC isolates that expressed podoplanin caused a significant decrease in absorbance in PRP, while podoplanin‐negative UCMSC did not (Fig. 3D). To further evaluate the mechanism, human recombinant CLEC‐2 protein was added to PRP and podoplanin positive UCMSC. CLEC‐2 protein significantly reduced the change absorbance compared to untreated controls (Fig. 3E), indicating competitive inhibition of platelet activation.

**Figure 4:**
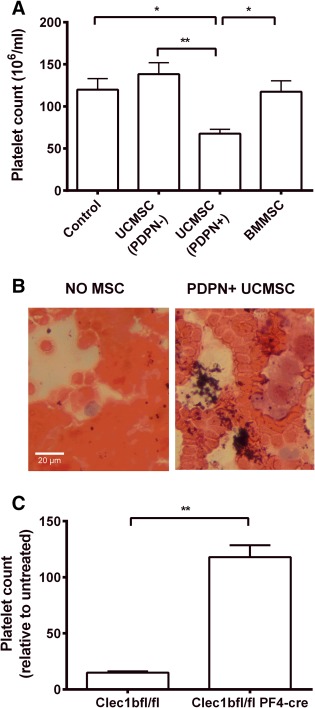
Effects of MSC on platelet counts in blood in vitro. **(A):** Whole human blood was stirred with podoplanin positive (PDPN+) UCMSC, podoplanin negative (PDPN−) UCMSC or BMMSC and platelet count was measured. Analysis of variance showed a significant effect of treatment on platelet count (*p* < .01). *, *p* < .05, **, *p* < .01 by Bonferroni test. Data are mean ± SEM from six experiments. **(B):** Representative images of H&E stained blood films made after the aggregation assay for blood alone or blood stirred with PDPN+ UCMSC using a bright‐field microscopy at ×40 magnification. **(C):** Blood from *clec1b* ^fl/fl^ or *clec1b* ^fl/fl^ PF4‐cre mice was mixed with PDPN+ UCMSC and platelet count was assessed and expressed relative to untreated blood. **, *p* < .01 by unpaired *t* test. Data are mean ± SEM from three experiments. Abbreviations: BMMSC, bone‐marrow mesenchymal stem cells; MSC, mesenchymal stem cells; UCMSC, umbilical cord mesenchymal stem cells.

Next, we mixed MSC with whole blood and measured the blood platelet count. The platelet counts were significantly reduced when blood was mixed with podoplanin‐positive UCMSC, but not with podoplanin‐negative UCMSC or with BMMSC (Fig. 4A). In blood smears platelets aggregates could be observed microscopically in the samples mixed with podoplanin‐positive UCMSC (Fig. 4B). We also added UCMSC to blood from control *Clec1b* ^fl/fl^ mice or mice deficient in CLEC‐2. Expression of CLEC‐2 on platelets from the latter was evaluated by flow cytometry and showed some variation; only those with <5% platelets expressing CLEC‐2 were analyzed further. There was a marked drop in platelet count when podoplanin‐positive UCMSC were added to blood from the control mice, but not when they were added to blood from mice deficient in platelet CLEC‐2 (Fig. 4C).

### Receptors Underlying UCMSC Adhesion to Platelets and in Whole Blood

**Figure 5:**
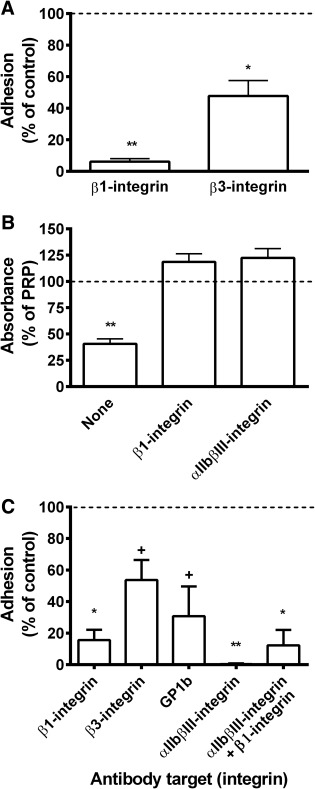
Roles of adhesion receptors in binding of umbilical cord mesenchymal stem cells (UCMSC) to platelets, induction of platelet aggregation by UCMSC or binding of UCMSC to collagen from whole blood. **(A):** Effect of pretreatment with antibodies against β1‐ or β3‐integrin on adhesion of UCMSC, suspended in culture medium, to platelet monolayers that had been deposited from whole blood onto fibronectin. Data are mean ± SEM from three experiments with adhesion expressed relative to untreated controls. *, *p* < .05 and **, *p* < .01 compared to untreated control by paired *t* test. **(B):** Effect of pretreating UCMSC with antibody against β_1_‐integrin, or treating PRP with antibody against αIIbβ3‐integrin, on aggregation of platelets after UCMSC were mixed with PRP. Data are mean ± SEM from three experiments with absorbance expressed relative to PRP alone. **, *p* < .01 compared to PRP alone by paired *t* test. **(C):** Effect of pretreating UCMSC with antibodies against β1‐integrin (*n* = 9) or β3‐integrin (*n* = 3), or of treating blood with antibody against GpIb (*n* = 3) or αIIbβ3‐integrin (*n* = 6) or both (*n* = 4) on adhesion of UCMSC perfused in whole blood over collagen. Data are mean ± SEM from n experiments expressed relative to adhesion for untreated controls. *, *p* < .05 and **, *p* < .01; +, borderline significance, *p* < .07; compared to untreated control by paired *t* test. Abbreviation: PRP, platelet‐rich plasma.

When UCMSC were perfused over a platelet monolayer deposited onto fibronectin, pretreatment with antibody against β1‐integrin caused >90% reduction in the number adherent; a smaller decrease was observed with antibody against β3‐integrin (Fig. 5A). When UCMSC were mixed with PRP, blocking β1‐integrin completely inhibited the decrease in absorbance (Fig. 5B). Pretreatment of platelets with antibody against αIIbβ3‐integrin also ablated the aggregation response (Fig. 5B). It was notable that microscopic observation of UCMSC added to PRP in the presence of anti‐αIIbβ3‐integrin did show single platelets attached to the MSC, indicating that direct adhesion of platelets to MSC was not through this receptor. Thus, β_1_‐integrins were required for interaction of UCMSC with platelets and platelet‐platelet aggregation operated through activation of αIIbβ3‐integrin.

When UCMSC were perfused over collagen in whole blood, adhesion was reduced by >80% when UCMSC were pretreated with antibody against β1‐integrin (Fig. 5C). Platelet deposition on the collagen was not modified, but the UCMSC did not adhere to the platelets. There was a lesser nonsignificant effect with antibody against β3‐integrin (Fig. 5C). We then tested the effect of treating platelets with antibodies against GpIb or αIIbβ3‐integrin. The former partially reduced platelet attachment to the surface and also adhesion of UCMSC (Fig. 5C). The latter left only a few single platelets on the collagen surface and completely abolished adhesion of UCMSC (Fig. 5C). In this case, the platelet‐coated MSC presumably could not bind direct to the exposed collagen. We tried blocking β_1_‐integrin on UCMSC in combination with blockade of platelet αIIbβ3‐integrin, which should leave “bare” MSC to flow across nearly “bare” collagen. A few single spherical MSC were observed to adhere, but the β_1_‐integrin blockade would also inhibit MSC adhesion to the collagen (Fig. 5C).

### Fate of MSC and Effect on Platelet Count When Infused into Mice

**Figure 6:**
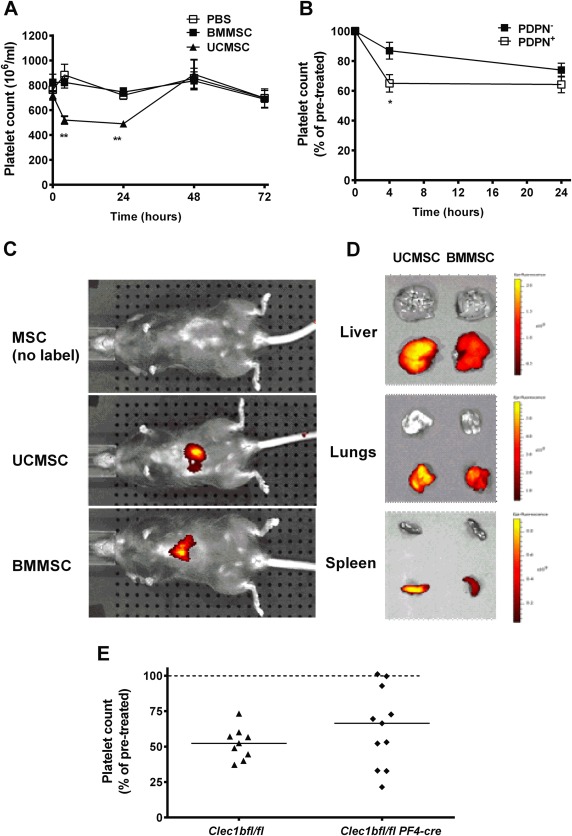
Fate of MSC infused into mice: effects on blood platelet counts and deposition in tissues. **(A):** Effects of infused MSC on blood platelet counts. Wild‐type mice were injected with phosphate‐buffered saline, UCMSC, or BMMSC, and platelet count measured before (*t* = 0) 4, 24, 48, or 72 hours after injection. Analysis of variance (ANOVA) showed a significant effect of treatment and of time (*p* < .01 in each case). **, *p* < .01 compared to PBS or to UCMSC by Bonferroni test. Data are mean ± SEM from at least 9 mice for *t* = 0, at least 6 mice for *t* = 4 and at least 7 mice for *t* = 24, and 2–4 mice at 48 or 72 hours. **(B):** Comparison of effects of podoplanin‐positive (PDPN+) or negative (PDPN−) UCMSC on blood platelet counts. Wild‐type mice were injected with PDPN+ MSC (*n* = 17) or PDPN− MSC (*n* = 12) and platelet count measured before (*t* = 0), 4 or 24 hours after injection. ANOVA showed that there is a significant effect of cell type and time (*p* < .01 in each case). *, *p* < .05 by *t* test compared to PDPN−. Data are mean ± SEM from (*n*) mice expressed as a percentage of the number of platelets before injection. **(C, D):** IVIS images of whole anesthetized mice 4 hours after injection with UCMSC or BMMSC (C), or of isolated liver, lungs, and spleen of two different mice 1 hour after injection (D). **(E):** Effects of infused podoplanin‐positive PDPN+ UCMSC on blood platelet counts in mice expressing or lacking CLEC‐2. *Clec1bfl/fl* mice (control, *n* = 9) or *Clec1bfl/fl PF4‐Cre* (*n* = 11) mice were injected with MSC, and platelet count measured before *t* = 0 and 4 after injection. Individual data (and median) are shown, expressed as a percentage of the number of platelets before injection. Abbreviations: BMMSC, bone‐marrow mesenchymal stem cells; MSC, mesenchymal stem cells; PBS, phosphate‐buffered saline; UCMSC, umbilical cord mesenchymal stem cells.

UCMSC, BMMSC, or PBS were injected into C57BL/6 mice via the tail vein. Injection of UCMSC caused a marked (∼40%) drop in circulating blood platelet count at 4 hours, which was maintained at 24 hours, but recovered by 48 hours (Fig. 6A). There were no significant changes in platelet count in mice that had received BMMSC or PBS. The decrease in platelet count was significantly greater after infusion of podoplanin‐positive UCMSC than podoplanin‐negative UCMSC (Fig. 6B). Fluorescently labeled MSC were imaged in live, anesthetized mice 4 and 24 hours after infusion, using the IVIS in vivo imaging system. For both cell types, dominant signals were observed from a location below the thorax suggesting that visceral organs such as liver and spleen contained major populations (Fig. 6C). Since the signal from organs in intact animals will inevitably be influenced by the optical properties of the overlying tissue, to increase sensitivity, major organs were retrieved after termination of the experiment at 1 or 24 hours after injection of MSC and imaged separately. Clear signals were evident from lung, liver, and spleen at both time points (e.g., Fig. 6D). There was no detectable difference in intensity of fluorescence between the UCMSC and BMMSC.

We next infused podoplanin‐positive UCMSC into the mice expressing or lacking CLEC‐2. As shown in Figure 6E, 4 hours after infusion, platelet count was reduced reliably in *Clec1bfl/fl* control mice. However, results were highly variable in the *Clec1bfl/fl PF4‐Cre* mice lacking CLEC2, with only 3 out of 11 mice maintaining an undisturbed platelet count (98% of preinfusion value).

## Discussion

We report the first studies of the adhesion of MSC from flow, including comparison of isolated cells with those in blood, and comparison of MSC from BM or UC. We made the surprising discoveries that both MSC types were captured by matrix proteins (collagen and fibronectin) more efficiently than by purified selectins, and that adhesion was massively altered when cells were perfused in blood. In blood, BMMSC barely adhered at all, while UCMSC bound to collagen, but not fibronectin, along with platelets in clumps. A key finding was that while both types of MSC bound to platelets, only UCMSC caused marked activation, driving platelet‐platelet aggregation. The difference was attributable to expression of podoplanin on UCMSC and not on BMMSC. Consequently, when injected into mice, podoplanin‐expressing UCMSC caused a marked reduction in platelet count, while BMMSC did not. Thus, when MSC are infused therapeutically, interaction with platelets is likely to influence their deposition into tissue, with expression of podoplanin adding a potential thrombotic effect.

Adhesion from flow may be a critical step for the recruitment of stem cells for vascular protection, although the mechanisms are still open to debate 13. One study found adhesion of BMMSC to endothelial cells at shear stress 0.1 Pa (equivalent to 140 s^−1^) 18, but we and others 8, 17 found that shear needed to be reduced to about 0.01 Pa (14 s^−1^) to allow capture to endothelial cells. Here, MSC could bind to matrix proteins, collagen, and fibronectin much more effectively than selectins. β_1_‐integrins played the major role for attachment of MSC to both proteins while for BMMSC β_3_‐integrins also contributed. Others have reported that BMMSC use β_1_‐integrin family members to bind to collagen and fibronectin under static conditions 29. Expression of adhesion molecules on MSC may be dependent on their source and the isolation method 30. However, we observed no difference in the surface expression of β_1_‐ and β_3_‐integrins that could account for UCMSC adhering more efficiently than BMMSC. Our results suggest that MSC would be more likely to adhere in a damaged vessel with exposed subendothelial matrix than in an inflamed vessel with intact endothelium.

**Figure 7:**
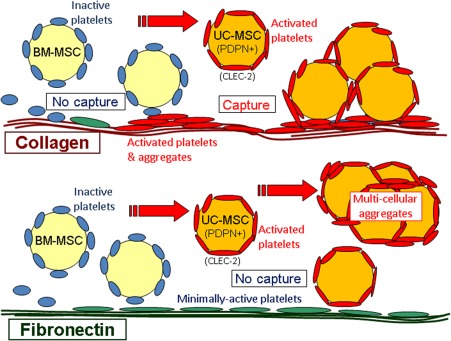
Interaction between MSC and platelets in blood and effects on adhesion when perfused over matrix proteins. In blood, BMMSC bind platelets which are inactive and effectively shield them so that they do not adhere to platelets deposited on fibronectin or collagen. UCMSC express podoplanin (PDPN) so that they not only bind platelets but also activated them through CLEC‐2. They do not adhere to the minimally activated platelets on fibronectin but do bind with activated platelets on collagen. In the flowing blood, the UCMSC with bound platelets may be able to drive wider‐spread activation and aggregation of platelets, that leads to a reduction in platelet count in vitro or in vivo. Abbreviations: BMMSC, bone‐marrow mesenchymal stem cells; MSC, mesenchymal stem cells; UCMSC, umbilical cord mesenchymal stem cells.

When added to blood, MSC were outnumbered by platelets by about 1,000:1. Both types of MSC may have attracted platelet satellites, but UCMSC must have induced extensive higher order aggregation in order to reduce platelet count. It seems that BMMSC coated with inactive platelets could not attach to the platelets on the fibronectin or collagen. However, UCMSC could activate bound platelets and then join in large structures on collagen, but not fibronectin where the “inactive” platelet monolayer did not capture them. For the UCMSC, adhesion to platelets was via β_1_‐integrins, with subsequent platelet aggregation supported by αIIbβ3‐integrin, which is known to mediate platelet‐platelet aggregation driven by a number of agonists 31. Since UCMSC bound both collagen and platelets through β_1_‐integrins, it was difficult to devise a blocking strategy that allowed direct binding of UCMSC to collagen in blood. While platelet attachment to collagen could be inhibited by blocking αIIbβ3‐integrin or GpIb, this left UCMSC in blood coated with platelets that could not bind to the collagen either. Combination of platelet and β_1_‐integrin blockade would leave collagen and the UCMSC surfaces vacant, but they would not be able to bind each other. These results are consistent with the scenario illustrated in Figure 7, where we propose a sequence of events if MSC injected into blood are exposed to collagen or fibronectin in damaged vessels.

In vivo, platelets facilitated BMMSC homing to lung vasculature in a rat model of pulmonary arterial hypertension 20. Intravital microscopy showed an association between infused MSC, leukocytes, and platelets at the site of lipopolysaccharide‐induced dermal inflammation in mice while platelet depletion decreased the trafficking of MSC to the site 19. Moreover, Langer et al. found that platelets attached to endothelial cells in vitro could increase BMMSC adhesion, and that injected BMMSC became attached to damaged arteries via platelets 32. In our experiments, during washout of blood, we observed small, round leukocytes rolling across platelets bound to collagen, and also some attached to the clumps of platelets and UCMSC. The numbers were not great enough to obstruct access of the MSC themselves to the collagen, but MSC may have facilitated localization of the leukocytes. The previous studies in vivo all used BMMSC. Our studies raised two questions—what might be the basis of the differences in interaction with platelets between BMMSC and UCMSC and what would happen, comparatively, if the two MSC were infused?

We did not observe marked differences in the locations of infused fluorescently labeled UCMSC or BMMSC tracked used live imaging. Most cells from either source were found in lungs, liver, and spleen. These observations are as expected from previous reports, showing, for example, early deposition in lungs after injection 33, 34, where sequestration may result from mechanical trapping of the large cells (∼20 µm diameter for our dissociated UCMSC or BMMSC). However, we did find that there were marked reductions in circulating platelet count when UCMSC were infused, but not BMMSC. This was consistent with the ability of UCMSC to reduce platelet count when mixed with human or mouse blood in vitro. Screening for differences in surface proteins on the MSC, we found that most UCMSC isolates expressed podoplanin, which is a ligand for the platelet receptor CLEC‐2 and known to activate platelets 25. Only the podoplanin‐positive UCMSC caused aggregation in PRP and a drop in platelet count in blood. Aggregation was reduced in the presence of soluble recombinant CLEC‐2, while platelet count was unaffected if UCMSC were mixed with blood from mice lacking CLEC‐2 expression. When UCMSC were injected into mice lacking expression of CLEC‐2, we found that the response in platelet count was highly variable compared to control mice; only 3 out of 10 mice showed no decrease, but 7 out of 12 knockout mice showed reductions between 30% and 70%. Thus, podoplanin‐CLEC‐2 interaction clearly drove platelet activation, aggregation, and reduction in count in blood in vitro. However, here, and to a lesser extent in other recent studies in our facility 35, the responses to podoplanin ligation in the *Clec1bfl/fl PFcre* model of CLEC2 reduction were variable in vivo. We checked expression of CLEC‐2 on platelets, and found less than 5% of platelets from *Clec1bfl/fl PFcre* mice to be positively labeled by antibody. Residual platelet CLEC‐2 may have induced an activation response through podoplanin on UCMSC, but there might also be another ligand for podoplanin active in vivo, or UCMSC may have had additional differences to the BMMSC which influenced platelet count in vivo.

Overall, functional studies used six different isolates of BMMSC (from a commercial source) and 10 isolates of UCMSC (derived in‐house). The isolates, their passage numbers, podoplanin status, and effects are listed in Supporting Information Table S1. Effects clearly correlate with podoplanin expression as discussed above. Passage number does not appear to be a factor in differences between BMMSC and UCMSC, or in podoplanin expression in UCMSC. Podoplanin expression remained stable in isolates, and after bimodal isolates were fractionated and returned to culture. The cause of variation in expression of podoplanin between UCMSC isolates is unresolved as donors were anonymous, but a systematic prospective study of this issue with appropriate ethical consent might be of interest. This may be important, because a number of reports have questioned compatibility of MSC with blood, where they may induce inflammatory or coagulation responses in vitro and if infused 36, 37, 38, 39. Those studies used BMMSC or adipose‐derived MSC (ADMSC), and indicated that responses increased with passaging and varied between sources, with ADMSC presenting tissue factor and inducing coagulation. BMMSC studied here did not express podoplanin at any passage, and the status of ADMSC is unknown in this context. UCMSC may have particular reaction with platelets, which depends on the donor but not evidently on passage number. It appears, however, that MSC for clinical use should be screened for interactions with blood elements that could influence safety of therapeutic infusion.

## Conclusion

The ability of MSC to differentiate into organ‐specific cells has led to their use for regenerative therapy 40. MSC are also able to suppress immune responses through effects of leukocytes and endothelial cells 6, 7, 8, 11, 26. Therapeutic use of MSC via infusion may depend on their ability to adhere in vessels of target organs and on interactions with platelets which may facilitate “homing” to inflamed tissue 19, 20, 32. We found that the origins of the MSC and their expression of podoplanin had impact on their behavior in the blood. Both MSC types studied here were able to bind platelets, which might facilitate attachment to damaged or inflamed vessels, although satellites formed by inactive platelets could “hide” the MSC in some circumstances. On the other hand, platelet activation by MSC in the circulating blood could cause thrombus formation and/or reduction in platelet count. There is thus a potential conflict between efficacy in localization and risk of thrombosis, associated with properties of MSC from different origins, which remains to be resolved.

## Author Contributions

L.S.: collection and/or assembly of data, data analysis and interpretation, manuscript writing, final approval of manuscript; A.A., L.S.C.W., C.W., H.M., and J.R.: collection and/or assembly of data, data analysis and interpretation, final approval of manuscript; M.A.: data analysis and interpretation, final approval of manuscript; S.P.W., P.N.N., N.K., and J.F.: conception and design, financial support, final approval of manuscript; G.E.R.: conception and design, financial support, data analysis and interpretation, final approval of manuscript; H.M.M. and G.B.N.: conception and design, financial support, collection and/or assembly of data, data analysis and interpretation, manuscript writing, final approval of manuscript.

## Disclosure of Potential Conflicts of Interest

The authors indicated no potential conflicts of interest.

## Supporting information

Additional Supporting Information may be found online in the supporting information tab for this article.

Supplementary Table 1
