# Supplementary material for: Origin‐Specific Adhesive Interactions of Mesenchymal Stem Cells with Platelets Influence Their Behavior After Infusion
Source: Stem Cells. 2018 Mar 23;36(7):1062–74. doi: 10.1002/stem.2811 (PMC6099218; doi:10.1002/stem.2811)
Supplement: Supplementary file 1 — Supplementary Table 1 [file STEM-36-1062-s001.docx]

**Supplementary Table 1**

**Characteristics and functional effects of MSC isolates used in the study.**

| **Isolate type and number^1^** | **Podoplanin expression** | **Passage number** | **Aggregation of PRP** | **Platelet count in blood (human)** | **Platelet count in blood (mice)** | **Platelet count in mice: Wild type** | **Platelet count in mice: CLEC2-/-** |
| --- | --- | --- | --- | --- | --- | --- | --- |
| BMMSC 4 | Negative | 8 | No effect | NT | NT | NT | NT |
|  |  | 9 | NT | NT | NT | NT | NT |
| BMMSC 5 | Negative | 7 | NT | NT | NT | No effect | NT |
|  |  | 8 | No effect | NT | NT | NT | NT |
|  |  | 9 | No effect | NT | NT | No effect | NT |
|  |  | 10 | NT | NT | NT | No effect | NT |
| BMMSC 6 | Negative | 6 | No effect | NT | NT | No effect | NT |
| BMMSC 7 | Negative | 5 | NT | No effect | NT | No effect | NT |
|  |  | 8 | No effect | NT | NT | No effect | NT |
| BMMSC 8 | Negative | 5 | NT | No effect | NT | NT | NT |
| BMMSC 10 | Negative | 5 | NT | No effect | NT | NT | NT |
|  |  | 6 | No effect | No effect | NT | NT | NT |
|  |  |  |  |  |  |  |  |
| UCMSC 5 | Bimodal | 6 | NT | NT | NT | Reduced count | Reduced count in 3 mice; unaltered in 1 mouse |
|  |  | 7 | Absorbance reduced | NT | NT | Reduced count | Reduced count in 3 mice; unaltered in 1 mouse |
|  |  | 8 | Absorbance reduced | NT | NT | NT | NT |
|  |  | 9 | Absorbance reduced | NT | NT | NT | NT |
|  |  | 10 | Absorbance reduced | NT | NT | NT | NT |
| UCMSC 7 | Bimodal | 3 | NT | NT | NT | Reduced count | NT |
|  |  | 5 | Absorbance reduced | NT | NT | Reduced count | NT |
| UCMSC 10 | Positive | 6 | NT | NT | NT | Reduced count | NT |
|  |  | 7 | NT | NT | NT | Reduced count | NT |
| UCMSC 11 | Negative | 3 | NT | No effect | NT | No effect | NT |
| UCMSC 11 | Negative | 4 | NT | No effect | NT | NT | NT |
|  |  | 5 | No effect | No effect | NT | No effect | NT |
| UCMSC 12 | Positive | 6 | NT | Reduced count | NT | NT | NT |
| UCMSC 13 | Positive | 5 | NT | Reduced count | NT | NT | NT |
| UCMSC 14 | Positive | 6 | NT | Reduced count | NT | NT | NT |
| UCMSC 17 | Bimodal | 5 | NT | Reduced count | NT | NT | NT |
|  |  | 6 | NT | NT | NT | Reduced count | NT |
| UCMSC 18 | Positive | 4 | NT | Reduced count | NT | NT | NT |
|  |  | 5 | NT | NT | Reduced count | NT | NT |
|  |  | 6 | NT | Reduced count | NT | NT | NT |
|  |  | 7 | NT | NT | NT | Reduced count | Reduced count in 2 mice; unaltered in 1 mouse |
|  |  | 8 | NT | NT | NT | Reduced count | NT |
| UCMSC 19 | Positive | 4 | NT | NT | Reduced count | NT | NT |

NT =Not tested in this assay

1. Numbers refer to order purchased (BMMSC) or isolated (UCMSC). The 10 UCMSC isolates listed were used in experiments. Podoplanin expression was characterised in 4 further isolates not used in functional experiments. Earlier isolates of BMMSC and UCMSC were not analysed in this project.

2. BMMSC were supplied at passage 2 used at passage 5 to 10. UCMSC were isolated in house and used at passage3 to 10. Passages were 3-way splits, so that number of cell doubling at passage 3 was approximately 5 and at passage 10 was approximately 16.
